# Differential orientation effect in the neural response to interacting biological motion of two agents

**DOI:** 10.1186/1471-2202-10-39

**Published:** 2009-04-27

**Authors:** Masahiro Hirai, Ryusuke Kakigi

**Affiliations:** 1 Department of Integrative Physiology, National Institute for Physiological Sciences, 38 Nishigonaka, Myodaiji, Okazaki, 444-8585, Japan; 2 Japan Society for the Promotion of Science, Chiyoda-ku, Tokyo 102-8471, Japan; 3 Department of Physiological Sciences, The Graduate University for Advanced Studies (Sokendai), Hayama, Kanagawa 240-0193, Japan

## Abstract

**Background:**

A recent behavioral study demonstrated that the meaningful interaction of two agents enhances the detection sensitivity of biological motion (BM), however, it remains unclear when and how the 'interaction' information of two agents is represented in our neural system. To clarify this point, we used magnetoencephalography and introduced a novel experimental technique to extract a neuromagnetic response relating to two-agent BM perception. We then investigated how this response was modulated by the interaction of two agents. In the present experiment, we presented two kinds of visual stimuli (interacting and non-interacting BM) with two orientations (upright and inverted).

**Results:**

We found a neuromagnetic response in the bilateral occipitotemporal region, on average 300 – 400 ms after the onset of a two-agent BM stimulus. This result showed that interhemispheric differences were apparent for the peak amplitudes. For the left hemisphere, the orientation effect was manifest when the two agents were made to interact, and the interaction effect was manifest when the stimulus was inverted. In the right hemisphere, the main effects of both orientation and interaction were significant, suggesting that the peak amplitude was attenuated when the visual stimulus was inverted or made to interact.

**Conclusion:**

These results demonstrate that the 'interaction' information of two agents can affect the neural activities in the bilateral occipitotemporal region, on average 300 – 400 ms after the onset of a two-agent BM stimulus, however, the modulation was different between hemispheres: the left hemisphere is more concerned with dynamics, whereas the right hemisphere is more concerned with form information.

## Background

Our visual system can extract much information on human actions from very limited cues. Biological motion (BM) is the phenomenon whereby one can perceive vivid action with just a dozen point-lights attached to the joints [1]. Previous studies on BM perception have mainly used only a single agent and have revealed that we can extract rich information from point-lights motion, such as identification of an individual [2], gender [3] or emotional state [4]. Interestingly, instead of using a single agent BM stimulus, a recent behavioral study used a 'two-agent' BM stimulus and demonstrated that synchronization of two agent interaction enhances the performance of point-light motion detection [5]. This finding indicates that a higher level of visual motion information, such as 'meaningful interaction' of two agents, can affect the detection sensitivity of BM. How then, is a unique visual stimulus, such as 'interaction' of two-agent information, represented in our neural system? Would this neural activity be modulated whether the two agents are interacting or not?

In a behavioral study using a single agent BM stimulus, at least two types of inversion effect for BM perception were demonstrated [6]. One was a shape-based inversion effects [7,8], which can be closely related to the face or body inversion effect [9]. Another was a motion-based inversion effect which is independent of shape processing, but depends only on the motion of the feet to detect the direction of a BM stimulus. These results imply that not only the shape from point-light motion information, but also the motion information itself can affect BM perception. Of course, these findings are based on results using just a single agent BM stimulus, however, this concept (i.e. shape-based and motion-based inversion effects) might apply for a two-agent BM stimulus.

Based on these frameworks, we introduced a two-agent BM stimulus (interacting or non-interacting) and tried to reveal the neural representation of the interaction information by inverting the visual stimulus. Prior to the experiment, we hypothesized that if the two-agent BM is dominated by form processing, we would expect a general inversion effect, which is independent of whether or not the two actors interact (i.e. form-based inversion effect). But if the neural system was sensitive to the dynamics of action between the two agents, then a significant interaction effect would be observed between the 'interaction' state of the agents and the stimulus orientation (i.e. motion-based inversion effect).

Since we can perceive human action with only a brief presentation of animation [10], it would be interesting to reveal the temporal profile of the neural response to 'interaction' information of two agents. To verify the above hypothesis and to clarify the temporal profile of the neural response to interacting information of a two-agent BM stimulus, we used magnetoencephalography (MEG) and introduced a novel experimental paradigm, 'double stimulus presentation', to extract a neuromagnetic response relating to BM perception (see Methods). We then evaluated how this component was modulated by manipulating interaction and orientation factors. The present study is the first attempt to reveal the neural activities relating to the interaction information between agents.

## Methods

### Participants

Nine healthy volunteers (one female and eight males; aged 28.1 ± 3.1, Mean ± S.D.) participated in experiments. All participants had normal or corrected-to-normal visual acuity. All participants provided informed consent for the experimental protocol, which was approved by the Ethics Committee of the National Institute for Physiological Sciences.

### Stimuli

Actors were recruited from our Institute. They have experience in boxing or Muay Thai and were asked to fight each other. Thirteen point-light markers were attached to each actor as previously described [5]. The point-lights motion was captured by a 3D motion capture system (Frame-DIAS IV, DKH Co., Ltd., Japan). After recording, we digitized the location of each point-light marker semi-automatically (the point-light maker was partly tracked by the experimenter), and we tracked the coordinates of each point-light marker in 2D (x-y) coordinates. We tracked 37 s of fighting and digitized each coordinate at 30 Hz.

**Figure 1:**
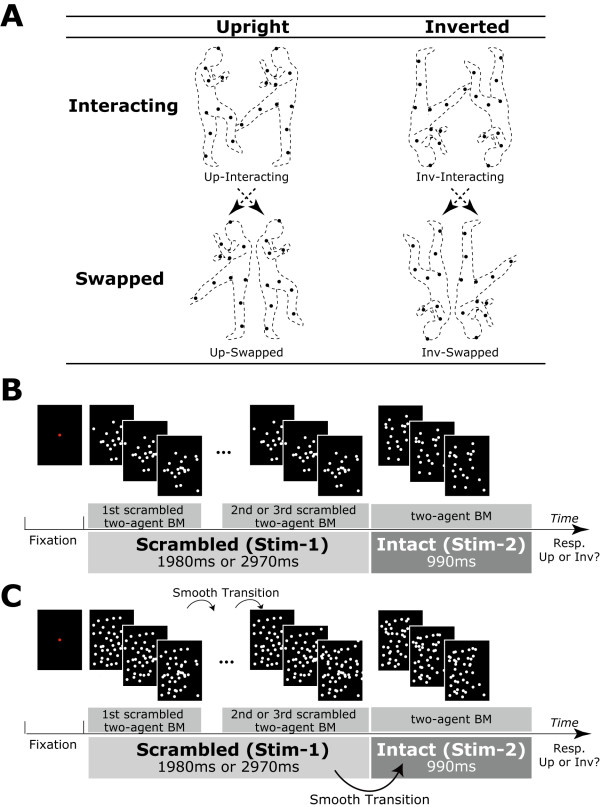
**Experimental stimulus, procedure**. (A) Experimental conditions. The visual stimulus consisted of the point-light actions of two agents (interacting and swapped) with two orientations (upright and inverted). In the interacting condition, the two agents interacted with each other. In the swapped condition, the positions of each agent were swapped with each other, thus participants perceived that each agent acted independently. (B) Experimental procedure (an example of Up-Interacting condition). For Stim-1, the scrambled two-agent BM (initial position of point-lights was spatially scrambled) stimulus was presented for 1980 or 2970 ms. For Stim-2, the two-agent BM stimulus was presented for 990 ms. Then, participants were required to judge whether the presented Stim-2 was upright or inverted. Note: to understand the stimulus more easily, the background static dots are not shown here in the Stim-1 and Stim-2 phases. However, in the actual experiment, the background static dots were superimposed on the point-light motion stimulus in both Stim-1 and Stim-2 phases, as shown in (C). Thus participants perceived a point-light animation consisting of white point-light dots. Due to the presence of background static point-light dots, participants could perceive a smooth point-light animation.

For the stimulus, we segmented the 37 s of digitized coordinates into 990 ms fragments. In this configuration, participants clearly recognized that two agents were fighting each other. For the interacting condition (Interacting-BM), the stimulus sequence was identical with the recorded data. For the swapped condition (Swapped-BM), the spatial positions of the agents were swapped with each other, thus the two agents appeared to be acting independently. It should be noted here that both interacting and swapped animations consisted of the identical number of point-lights and identical motion vectors, the only difference was the spatial position of each agent. As shown in Figure 1A, we manipulated the orientation of each stimulus. As a result, four kinds of visual stimuli were produced (Up-Interacting, Up-Swapped, Inv-Interacting, and Inv-Swapped).

### Design and Procedure

We applied a 'double stimulus presentation' method to extract a visually evoked field (VEF) response relating to two-agent BM processing, as described in previous studies [11,12]. The stimulus sequence consisted of two phases: as shown in Figure 1B. We used a scrambled two-agent BM as a prestimulus (Stim-1) and an intact two-agent BM as a target stimulus (Stim-2) to attenuate the onset- and motion-related VEF response to Stim-1, because it is considered that the neuronal system responding to the onset- or motion-related activity is habituated. In all conditions, Stim-1 was presented for 1980 or 2970 ms by presenting the scrambled two-agent BM stimulus twice or three times repeatedly; then, intact two-agent BM (Stim-2) was presented immediately for 990 ms. Thus the participants perceived swaying point-lights that transiently formed a two-agent fighting point-light stimulus.

To attenuate the onset- and motion-related VEF responses in Stim-2, we needed to present the scrambled two-agent BM stimulus repeatedly. Because the two-agent BMs were not periodic actions, like the point-light walker (PLW) developed by Cutting [13], the positions of the point-lights in the initial frame were quite different from the positions of the point-lights in the end frame. Thus we cannot present the animation smoothly when the stimulus is simply presented during Stim-1. To present a smooth animation, we superimposed four kinds of static point-lights onto the two-agent BM animations (Figure 1C): static point-lights of the initial and final frames of the two-agent BM stimuli. As a result, participants could smoothly perceive the repeated point-light action, as if the point-lights appeared and disappeared among crowded static point-lights (Figure 1C). Since we superimposed the static point-lights on the point-light motion stimulus, it was hard for participants to distinguish the interacting stimulus from the swapped stimulus at the presentation of the initial frame of the point-light stimulus. Consequently, the total number of point-lights was 130, including the point-light motion stimulus (interacting or swapped). The spatial distribution of point-lights in the initial frame was identical across conditions.

Animations were displayed subtending a visual angle of approximately 3 × 3° on a projector screen at a viewing distance of 200 cm. All points were white (38.4 cd/m^2^) against a black background (6.5 cd/m^2^). A red fixation point was presented at the center of the screen throughout the experiment. The experiment consisted of six blocks. In each block, each experimental condition (Up-Interacting, Up-Swapped, Inv-Interacting, and Inv-Swapped) was randomly presented 15 times. In total, each conditional stimulus was presented 90 times. To maintain their attention at the center of the screen, participants were instructed to press a button at each trial to indicate whether each presented intact stimulus (Stim-2) was upright or inverted.

After MEG experiments, behavioral experiments were performed where participants were required to report the impression of the interaction strength on an 11-point scale (0 = none, 10 = strongly interacting). The experimental settings for the behavioral experiments were identical to those of the previous MEG experiment. Each visual stimulus was presented three times and participants were then required to report the score of the visual stimuli. Participants evaluated the visual stimulus once per visual stimulus. The order of visual stimulus presentation was randomized across participants.

### MEG recording and Data analysis

VEFs were recorded using a helmet-shaped 306-channel detector array (Vectorview, ELEKTA, Neuromag, Helsinki, Finland), which comprised 102 identical triple sensor elements. Each sensor element consisted of two orthogonal planar gradiometers and one magnetometer coupled to a multi-SQUID (superconducting quantum interference device), thus providing three independent magnetic field measurements. We analyzed the MEG signals recorded from the 204 planar-type gradiometers, as described previously [14] because the signals from these planar sensors are strongest when the sensors are located just above local cerebral sources [15]. Eye position was also monitored using an infrared eye tracker (Iscan Pupil/Corneal Reflection Tracking System, Cambridge, MA) and trials contaminated by eye movements (> 0.5°) or blinks were rejected. MEG signals were recorded with 0.1 – 100 Hz band-pass filters and digitized at 500 Hz.

#### Data analyses (MEG data)

In off-line analyses of MEG recordings, a 0.1 – 30 Hz band-pass filter was applied [16] and the signals evoked by six visual stimuli (two kinds of Stim-1 and four kinds of Stim-2) were averaged separately. For scrambled stimulus (Stim-1), identical visual stimuli were used in both interacting and swapped conditions, therefore, we merged Up-interacting and Up-swapped into upright condition, and Inv-interacting and Inv-swapped condition into inverted condition. Trials in which the MEG signal variation exceeded 3000 fT/cm were discarded. The analysis window was extended for 1000 ms following the onset of both Stim-1 and Stim-2. A prestimulus period of 200 ms was used as the baseline for both Stim-1 and Stim-2. The baseline period for Stim-2 was defined as the 200 ms before the offset of Stim-1.

We calculated areal mean (AM) signals of (i) twenty gradiometer pairs over the left occipitotemporal region and (ii) twenty gradiometer pairs over the right occipitotemporal region to cover the evoked neuromagnetic responses to Stim-1 and Stim-2 across participants. We first computed vector sums by squaring the MEG signals of each gradiometer pair, summing these signals together and then calculating the square root of this sum. The AM signals were computed by averaging these vector sums for each area of interest (left and right occipitotemporal region). The AM signals were computed individually for each subject. Finally, we calculated overall group averages [17-19]. The peak amplitude and latency of the component was determined for each subject by evaluating a 300 ms window centered on the 400 ms after stimulus onset, based on a previous study [11]. The peak amplitude and latency of prominent responses in the VEF waveform were then measured at the peak responses.

For the behavioral data, the correct performance and the score of the interaction strength was subjected to a two-way analysis of variance (ANOVA) with orientation (upright, inverted) and interaction (interacting, swapped) as factors. For the VEFs elicited by scrambled stimulus (Stim-1), the peak amplitudes and latencies were subjected to a two-way ANOVA with hemisphere (left, right) and orientation (upright, inverted) as factors. For the VEFs elicited by intact stimulus (Stim-2), the peak amplitudes and latencies were subjected to a three-way ANOVA with hemisphere (left, right), orientation (upright, inverted) and interaction (interacting, swapped) as factors. If the sphericity assumption was violated in Mauchly's sphericity test, then the Greenhouse-Geisser correction coefficient epsilon was used to correct the degrees of freedom, and then the *F *and *P *values were recalculated. We considered statistical significance as *p *< 0.05.

## Results

### Behavioral Data

**Figure 2:**
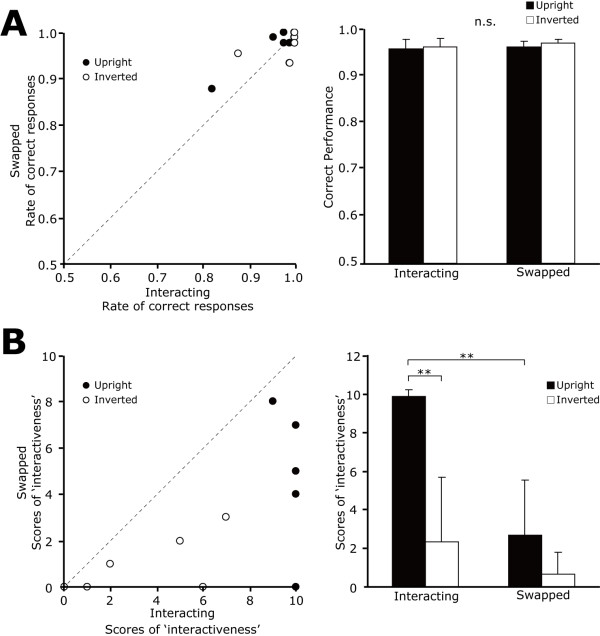
**Behavioral results of each visual stimulus**. (A) Left: scatter plot of individual behavioral performances during MEG experiments. The horizontal line indicates the rate of correct response for the interacting condition and the vertical line indicates the rate of correct response for the swapped condition. Each symbol indicates the stimulus orientation (solid: upright, open: inverted). Right: averaged rates of correct responses for each stimulus. Black bar indicates the upright stimulus and the white bar indicates the inverted stimulus. Error bars indicate the standard deviation (S.D.). (B) Left: scatter plot of individual behavioral scores of interaction strength for each visual stimulus. As in (A), the horizontal line indicates the score of interaction strength for the interacting condition and the vertical line indicates the interaction strength for the swapped condition. Right: averaged scores for each stimulus. Error bars indicate the S.D. For both scatter plots, identical results were obtained from several participants, thus the marks overlap. As a result, the number of displayed marks is less than nine.

Figure 2A shows the individual correct performances (left) and the averaged results (right). No significant difference among the correct performances was observed (*Fs *< 1.2, *ps *> 0.3). In all conditions, the average correct performance was above 94%, indicating that the participants attended the screen and could correctly judge the orientation of the visual stimuli.

After MEG experiment, participants were required to report the impression of the interaction strength on an 11-point scale. The individual data are shown in Figure 2B (left) and the averaged values are shown in Figure 2B (right). The value of each score is as follows: Up-Interacting (9.9 ± 0.3; mean ± S.D.), Up-Swapped (2.7 ± 3.4), Inv-Interacting (2.3 ± 2.9) and Inv-Swapped (0.7 ± 1.1). The results showed that the main effect of orientation [*F*(1,8) = 45.6, *p *< 0.01] and interaction [*F*(1,8) = 49.5, *p *< 0.01], and orientation × interaction were significant [*F*(1,8) = 13.5, *p *< 0.01]. Subsequent analysis revealed that the score of interaction strength in the upright condition was significantly larger compared with that in the inverted condition when the two agents were interacting [*F*(1,16) = 53.3, *p *< 0.01; 9.9 ± 0.3 vs. 2.3 ± 2.9, Mean ± S.D.]. Moreover, the score of interaction-strength in the interacting condition was significantly larger compared with that in the swapped condition when the stimulus was upright [*F*(1,16) = 53.8, *p *< 0.01; 9.9 ± 0.3 vs. 2.7 ± 3.4].

### MEG Data

**Figure 3:**
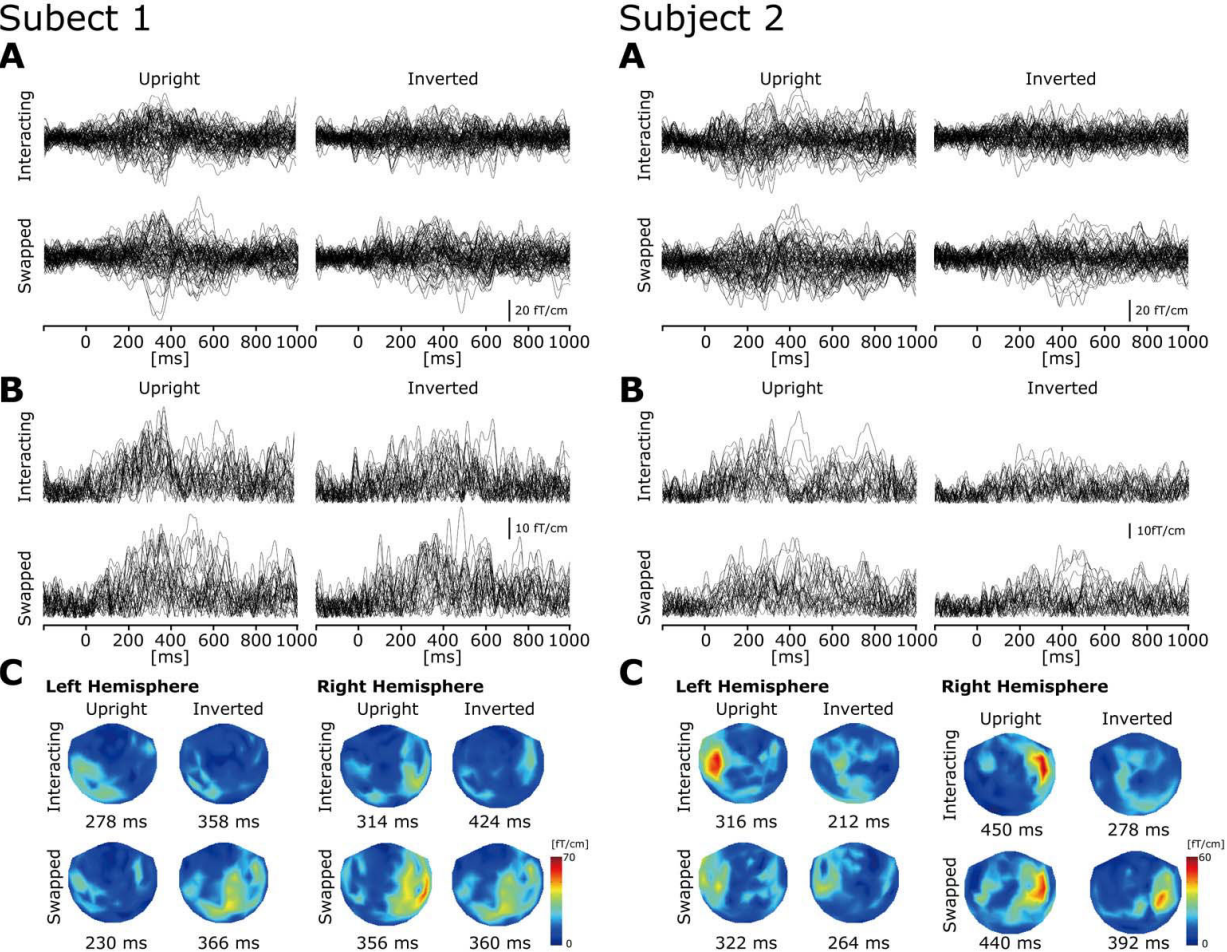
**Individual MEG waveforms (2 subjects)**. Individual data for subject 1 (left panel) and subject 2 (right panel). (A) raw MEG waveforms (recorded from gradiometers) from the bilateral occipitotemporal sensors (80 raw waveforms were superimposed in total). (B) vector sum waveforms (calculated by squaring the MEG signals of each gradiometer pair, summing these signals together and then calculating the square root of this sum) from the bilateral occipitotemporal sensors (40 waveforms were superimposed in total). (C) isocontour maps at the peak latency of the areal mean (AM) waveforms in each hemisphere for each intact visual stimulus (Stim-2). The prominent responses were mainly observed in the occipitotemporal sensors.

**Figure 4:**
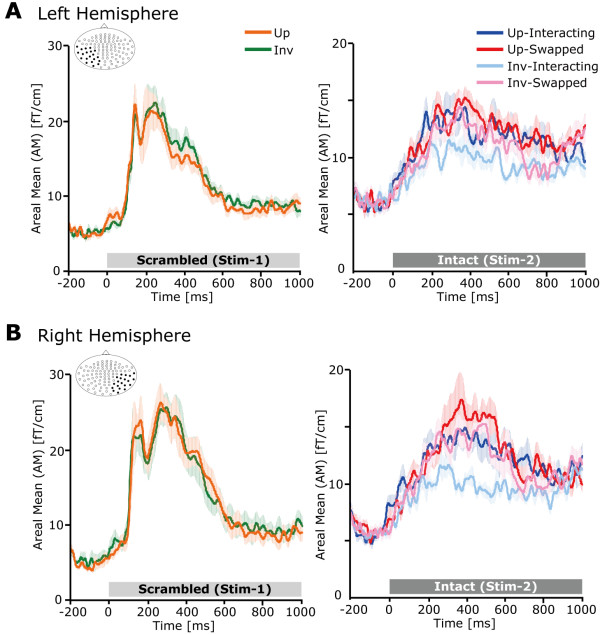
**MEG responses to Stim-1 and Stim-2**. Grand averaged Areal Mean (AM) waveforms (N = 9) in the (A) left hemisphere and (B) right hemisphere. Left panel: orange line indicates the upright scrambled two-agent BM (Stim-1) and the green line indicates the inverted scrambled two-agent BM (Stim-1). Right panel: blue line indicates the Up-Interacting condition, red line indicates the Up-Swapped condition, aqua line indicates the Inv-Interacting condition, and pink line indicates the Inv-Swapped condition. We computed areal means across 20 gradiometer pairs in left and right occipitotemporal regions, respectively (the location of selected sensors is shown in the left upper panel of each graph). While a neuromagnetic response was observed at 250 – 270 ms on average in Stim-1, a neuromagnetic response was observed at 340 ms on average in Stim-2. The shaded area in the background depicts the standard error of the mean (S.E.M.).

**Figure 5:**
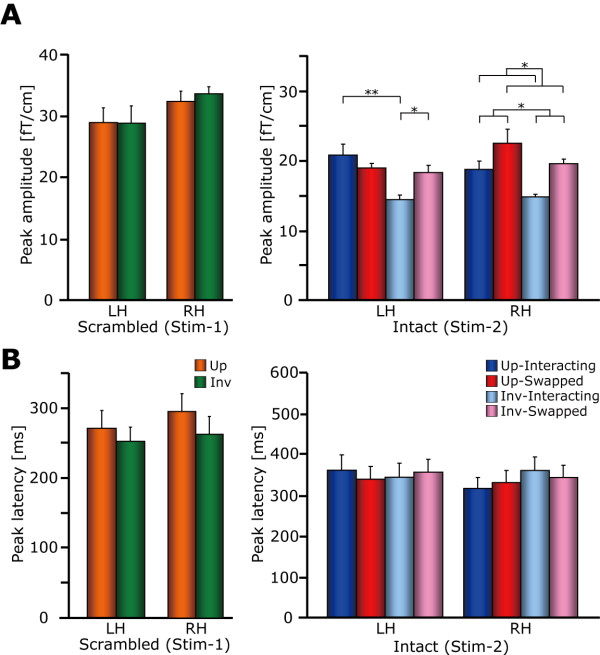
**Peak amplitude and latency**. (A) Peak amplitude in Stim-1 (left) and Stim-2 (right). For Stim-1, no significant effect was observed. For Stim-2, the 3-way interaction was significant. Subsequent analysis revealed that both orientation and two-agent interaction effect was significant in the right hemisphere. In the left hemisphere, 2-way interaction between orientation and two-agent interaction was significant (see text). (B) Peak latency in Stim-1 (left) and Stim-2 (right). No significant effect was observed for either Stim-1 or Stim-2. Error bar indicates the standard error of the mean (S.E.M.). * *p *< 0.05, ** *p *< 0.01.

Representative individual MEG responses to intact two-agent stimulus (Stim-2) are shown in Figure 3. Prominent neuromagnetic responses were mainly found at 200 – 450 ms in the bilateral occipitotemporal regions. The grand-averaged waveforms are shown in Figure 4. For Stim-1, neither the peak amplitude nor latency was significantly different between upright and inverted conditions in both hemispheres [peak amplitude: *Fs *< 2.6, *ps *> 0.2, peak latency: *Fs *< 3.3, *ps *> 0.1] (Figure 5, left). For the peak amplitude in Stim-2 (Figure 5A, right), the 3-way interaction was significant [*F*(1,8) = 5.9, *p *< 0.05]. We then carried out two partial 2-way ANOVA analyses for each hemisphere. For the left hemisphere, the 2-way interaction was significant [*F*(1,8) = 12.5, *p *< 0.01], suggesting that the peak amplitude induced by the upright condition was significantly larger compared with that of the inverted condition when the two agents were interacting [*F*(1,16) = 28.4, *p *< 0.01; 20.8 ± 1.5 vs. 14.4 ± 0.6 fT/cm, Mean ± S.E.]. Moreover, the peak amplitude induced by the swapped condition was significantly larger compared with that of the interacting condition when the stimulus was inverted [*F*(1,16) = 10.6, *p *< 0.01; 14.4 ± 0.6 vs. 18.3 ± 1.0 fT/cm]. However, neither the orientation effect in the swapped condition [*F*(1,16) = 0.3, *p *= 0.59] nor the interaction effect in the upright condition [*F*(1,16) = 1.3, *p *= 0.14] was significant.

**Figure 6:**
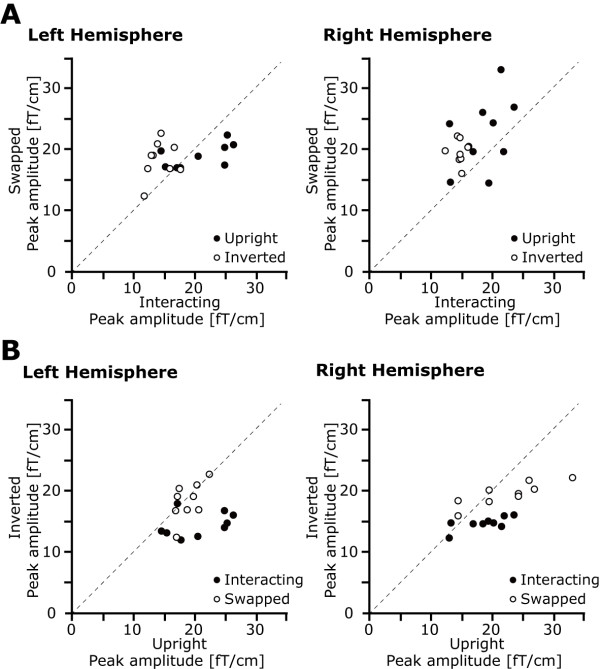
**Individual peak amplitude (Stim-2)**. Scatter plots of individual peak amplitudes for each visual stimulus (Stim-2). (A) The horizontal line indicates the peak amplitude for the interacting condition and vertical line indicates the peak amplitude for the swapped condition. Each symbol indicates the stimulus orientation (solid: upright, open: inverted). (B) The horizontal line indicates the peak amplitude for the upright condition and vertical line indicates the peak amplitude for the inverted condition. Each symbol indicates the stimulus condition (solid: interacting, open: swapped).

For the right hemisphere, the main effects of orientation [*F*(1,8) = 8.8, *p *< 0.05] and interaction [*F*(1,8) = 11.0, *p *< 0.05] were significant. This suggests that the peak amplitude induced by the upright condition was significantly larger compared with that induced by the inverted condition (20.7 ± 1.2 vs. 17.3 ± 0.7 fT/cm). Moreover, the peak amplitude induced by the swapped condition was significantly larger compared with that induced by the interacting condition (21.1 ± 1.1 vs. 16.8 ± 0.8 fT/cm). Other partial 2-way ANOVAs did not reveal significant inter-hemispheric differences. For the peak latency (Figure 5B, right), no significant effects were observed [*Fs *< 2.1, *ps *> 0.2]. To clarify the individual variation of the peak amplitudes, scatter plots are also shown in Figure 6.

## Discussion

In the present study, we used a two-agent BM stimulus and investigated how the 'interaction' information of two agents was represented in our neural system. The behavioral result revealed that the rating of the interaction strength was reduced for the inverted visual stimulus compared with the upright visual stimulus when the two agents were interacting, but such an orientation effect was not observed in the swapped condition. In the MEG results, we observed a VEF peak response to the intact stimulus (Stim-2) in the bilateral occipitotemporal region after an average of 300 – 400 ms. For the peak amplitude in the left hemisphere, a two-way interaction between the 'interaction' state of the two-agents and the stimulus orientation was significant. It suggests that the orientation effect was manifest when the two agents were made to interact, and that the interaction effect was manifest when the stimulus was inverted. In the right hemisphere, however, we did not find a significant interaction between the 'interaction' state of the two-agents and stimulus orientation. Instead, the main effect of both orientation and interaction was significant, suggesting that the activity is higher for the upright than for the inverted stimulus. However, activity is lower for interactive than for swapped displays. These results demonstrated that the 'interaction' information of two agents can affect the neural activities in the bilateral occipitotemporal region, however, the modulation can be different between hemispheres. In light of our hypothesis, the processing mechanisms of two-agent 'interaction' information in the left hemisphere can be explained predominantly by the motion-based inversion effect, and in the right hemisphere the mechanisms can be predominantly explained by the form-based inversion effect, with partial involvement of the motion-based inversion effect (see below).

In the left hemisphere, a 2-way interaction between orientation and interaction was significant. This indicates that the inversion effect was observed when the two agents interacted, and that the effect of the two-agent interaction was significant when the visual stimulus was inverted. This result seems to be compatible with a previous behavioral study [5]. Neri et al. reported that observers displayed significantly higher noise tolerance for a synchronized two-agent BM stimulus than for a desynchronized BM stimulus. However, this increased noise tolerance for the synchronized BM stimulus was not observed when the stimulus was inverted. Despite the fact that the visual stimulus, the experimental paradigm and the indexes (i.e. the number of noise dots vs. neuromagnetic responses) were different from the present experiment, the effect of interaction between agents were commonly observed. Thus it is natural to postulate that the meaningful interaction between two agents, or more specifically, the meaningful point-light motion of limbs between two agents can modulate neural activities. Interestingly, not only for a two-agent BM stimulus and also for a single BM stimulus, the importance of local motion, particularly the information of the feet, has been reported in the detection of PLW direction [6]. The authors argued that there is another inversion effect that depends on the motion of the feet, which is independent of shape. It would seem possible that such local motion detectors exist, not only in the visual processing of a single BM stimulus, but also for the processing of a two-agent BM stimulus.

In the right hemisphere, we did not observe a two-way interaction as in the left hemisphere, but each main effect of orientation and interaction was significant: the activity is higher for upright than for inverted stimuli, but then it is also lower for interactive compared with swapped displays. For a significant orientation effect, the result is in line with previous neuroimaging studies (e.g. [20]). It was also revealed that the neural activities were significantly attenuated for the inverted BM stimulus compared with the upright BM stimulus. In the light of our initial hypothesis, it is likely that the modulated neural activities in the right hemisphere can be predominantly explained by the form-based inversion effect.

One might think that the present results for the orientation effect could be considered somewhat inconsistent with previous ERP and MEG studies [11,21]: the amplitudes of the component that was related to BM perception in both upright and inverted stimuli were not significantly different. This discrepancy could be due to the complexity of the visual stimulus used in the present experiment. In the previous studies, a PLW stimulus was introduced and presented many times to obtain evoked responses. Thus, even in the inverted condition, participants could easily perceive the figure of a walking human from point-lights motion. However, in contrast to the PLW stimulus, the present stimulus is much more complex and the visual stimulus was presented with a number of static point-lights. Thus it was hard to interpret what was really represented for the inverted version of the visual stimulus and the neural activities could be attenuated by the inversion of the stimulus. This speculation was based on the result of our preliminary experiment; it was hard for some participants to understand what the inverted version of the two-agent BM stimulus was, even when the stimulus was presented many times.

Interestingly, in addition to the orientation effect, we also found a significant main effect of interaction for both upright and inverted stimuli. This is a somewhat unexpected result, and it seems difficult to explain. It implies that the global motion pattern information of two agents (not restricted to local motion, such as limb point-lights motion) might modulate the neural activities in the right hemisphere. One plausible explanation is that the modulatory activities might reflect the 'naturalness' of the agent interaction. In the interacting condition, the two-agents' point-light movement is natural, whereas in the swapped condition the movement seems to be somewhat unnatural in terms of the 'meaningful' interaction. Such unnatural movement might modulate the neural activities. Of course this interpretation is rather speculative and further studies should address this point. Here, we should only mention that the 'interaction' information can be coded differently between the interacting and the swapped conditions, irrespective of orientation of the visual stimulus. Based on these results, it is likely that the modulated neural activities in the right hemisphere can be predominantly explained by the form-based inversion effect, but that the motion-based inversion effect might also be partly involved.

It should be noted that each agent was identical in both interacting and swapped conditions (the number of point-lights, local motion information and the size of visual stimulus), thus it was hard to explain that the differential neural activities were simply due to the low-level visual features. Rather, the modulated neural activities may be due to the motion pattern of point-lights of the agents' legs or arms that contain meaningful information of human interaction [5]. Of course, we cannot currently specify what kinds of motion information modulate the neural activities, but we can at least provide direct electrophysiological evidence for the differential neural response between interactive and non-interactive two agent visual stimuli.

The observed interhemispheric differences in the neuromagnetic responses to two-agent BM might be related to the differential visual processing, as previously reported [22]: the dominance of local information processing in the left hemisphere and the dominance of global information processing in the right hemisphere [23]. The present findings seem to be compatible with this view. That is, the neural activities in the left hemisphere were more sensitive to the interaction between two agents (i.e. local motion information, such as agents' limb movement), whereas the neural activities in the right hemisphere were more sensitive to the global shape from point-light motion or global motion pattern information. Even at a single agent level, asymmetric oscillatory activities have also been reported during perception of a PLW stimulus [24]. Pavlova et al have argued that a stronger left-side enhancement in the oscillatory response over the occipital cortices is likely to reflect the early processing of a coherent structure emerging from the array of moving dots, while a late right-side increase over the temporal areas may reflect the processing of the whole configuration. Of course, we did not directly associate asymmetric oscillatory activities with the present evoked responses, however, such functional asymmetries might exist, even in the visual processing of a two-agent BM stimulus.

By using an identical experimental procedure, our previous study demonstrated that the source of the neuromagnetic response to a single PLW stimulus was mainly located in the vicinity of the posterior superior temporal sulcus (pSTS) [11]. Several neuroimaging studies have demonstrated that the superior temporal sulcus (STS) was not only implicated in action processing [25-27], but also in the detection of animacy, induced by the motions of simple geometrical objects [28,29]. Thus it is possible that the observed activities reflect cortical activities in the vicinity of the STS region. However, as Neri et al. pointed out [5], the processing of interaction information can be performed in a larger network of cortical areas, spanning both the STS and the mirror neuron system (MNS). Action interpretation is thought to result from an implicit simulation process [30], thus it is possible that the modulatory activities between interacting and swapped conditions in this occipitotemporal region are the output of the feedback from the MNS [31]. Of course, we cannot confirm whether this is really the case and further studies should address the possibility of MNS involvement.

In the present experiment, we introduced a 'double stimulus presentation' method; thus the neural responses relating to onset- or motion-related responses would be observed in Stim-1 and would be attenuated in Stim-2. This is supported by the result in Stim-1. That is, we could not find any significant orientation effect in the neuromagnetic response to Stim-1. Moreover, consistent with previous MEG studies for motion perception, the peak latency of the Stim-1 response was observed at 200 – 250 ms on average [12,32]. Thus it is plausible to interpret that the observed VEF responses during Stim-1 reflect motion processing, as in previous MEG studies, and that the VEF responses during Stim-2 reflect two-agent BM processing extracted from the point-lights motion.

This result might contribute to the refinement of the BM processing model, as proposed by Troje [33]. This model consisted of several different processing layers (life detection, structure-from-motion, action recognition, and style recognition). The processing layers were mainly designed to explain single agent BM processing. Here, another processing layer, such as a multi-agent BM processing layer, might be added to these processing layers. In this layer, processing mainly focused on the relationship between agents rather than single agent processing, such as structure-from-motion and style recognition. This would make it sensitive to the dynamic relationship of the point-light motion of arms or legs between agents. Of course, this is speculation based on the present findings, thus further studies should be conducted to determine whether different levels of processing are involved in multi-agent BM processing.

## Conclusion

We used MEG and introduced a novel experimental technique to extract a neuromagnetic response relating to two-agent BM perception. We found that the component that was related to BM processing was modulated by orientation and the manner of two-agent interaction, and that the modulation was different between hemispheres. The left hemisphere is more concerned with dynamics (i.e. dominance of a motion-based inversion effect), whereas the right hemisphere is more concerned with form information (i.e. dominance of a form-based inversion effect). Moreover, it implies that another processing layer might be involved in the BM processing model proposed by Troje [33].

## Authors' contributions

MH conceived of the study, designed the experimental setup, acquired and analyzed the data. All authors participated the data evaluation and interpretation and in writing the manuscript and have approved the final version of the manuscript.
